
JOURNAL INFORMATION
==============================
NLM Title Abbreviation: Innov Aging
Journal ID: Innov Aging
NLM Journal ID: 101703706
Journal ID: innovateage

Innovation in Aging

EISSN: 2399-5300
Publisher: Oxford University Press

ARTICLE INFORMATION
==============================
PMCID: PMC6845712
DOI: 10.1093/geroni/igz038.893
Article ID: igz038.893
Article version: 1
Subjects: Session 1270 (Symposium)

PERSONALIZED AND ADAPTIVE VR/AR FOR OLDER ADULTS: EIGHT CRITICAL NEEDS

Gilbert Stephen B
Iowa State University , Ames, Iowa, United States
Publication date: 2019 Nov
Electronic publication date: 2019 Nov 8
Volume: 3
Issue: Suppl 1
Issue title: Program Abstracts from the GSA 2019 Annual Scientific Meeting “Strength in Age—Harnessing the Power of Networks”
First page: S239
Last page: S239
Copyright: © The Author(s) 2019. Published by Oxford University Press on behalf of The Gerontological Society of America.
Copyright year: 2019
License: This is an Open Access article distributed under the terms of the Creative Commons Attribution License (http://creativecommons.org/licenses/by/4.0/), which permits unrestricted reuse, distribution, and reproduction in any medium, provided the original work is properly cited.
License URL: https://creativecommons.org/licenses/by/4.0/

Page count: 1

==============================
### PERSONALIZED AND ADAPTIVE VR/AR FOR OLDER ADULTS: EIGHT CRITICAL NEEDS

Stephen B. Gilbert[1], *1. Iowa State University, Ames, Iowa, United States*

In the past decade there has been considerable progress in the technology associated with virtual reality and augmented reality, along with decreased costs. But with these advances come a new set of research questions. In July 2017, the National Science Foundation hosted a VR/AR visioning workshop to explore these challenges. The workshop focused on the research needed to achieve individual personalization and adaptation in the areas of visual, auditory and tactile perception, as well as the social, behavioral and cognitive patterns key to adaptation. These discussions yielded eight priorities for upcoming research for VR/AR. In this paper, we will describe these eight priorities from the perspective of the gerontology community, looking at the research gaps that must be addressed to apply VR/AR successfully with older adults.
